# From Flexible and Stretchable Meta-Atom to Metamaterial: A Wearable Microwave Meta-Skin with Tunable Frequency Selective and Cloaking Effects

**DOI:** 10.1038/srep21921

**Published:** 2016-02-23

**Authors:** Siming Yang, Peng Liu, Mingda Yang, Qiugu Wang, Jiming Song, Liang Dong

**Affiliations:** 1 Department of Electrical and Computer Engineering, Iowa State University, Ames, IA 50011.

## Abstract

This paper reports a flexible and stretchable metamaterial-based “skin” or meta-skin with tunable frequency selective and cloaking effects in microwave frequency regime. The meta-skin is composed of an array of liquid metallic split ring resonators (SRRs) embedded in a stretchable elastomer. When stretched, the meta-skin performs as a tunable frequency selective surface with a wide resonance frequency tuning range. When wrapped around a curved dielectric material, the meta-skin functions as a flexible “cloaking” surface to significantly suppress scattering from the surface of the dielectric material along different directions. We studied frequency responses of multilayer meta-skins to stretching in a planar direction and to changing the spacing between neighboring layers in vertical direction. We also investigated scattering suppression effect of the meta-skin coated on a finite-length dielectric rod in free space. This meta-skin technology will benefit many electromagnetic applications, such as frequency tuning, shielding, and scattering suppression.

Metamaterials have attracted considerable attention due to their inaccessible electromagnetic (EM) properties that can be hardly found in natural materials. The unique properties of negative permittivity, negative refractive index, and index close to zero^1^,^2^,^3^,^4^,^5^,^6^,^7^,^8^,^9^,^10^,^11^,^12^, allow metamaterials to be employed in many emerging applications such as sub-wavelength resolution imaging^13^,^14^, filtering^15^, and cloaking^16^,^17^,^18^,^19^. Compared to conventional microwave filters, metamaterial-based counterparts have demonstrated the potential to obtain compact sub-wavelength size and left-handed behaviors. By manipulating EM waves with metamaterials, invisibility cloaks capable of suppressing the wave scattering and/or guiding the waves around the hidden object in the microwave, terahertz and optical regimes have been theoretically and experimentally realized^16^,^17^,^18^,^19^,^20^,^21^,^22^,^23^,^24^,^25^,^26^,^27^,^28^. Different kinds of sub-wavelength resonators have been researched to achieve selective frequency responses. Among them, split ring resonator (SRR) is a widely proposed magnetic resonant structure^29^,^30^. While numerous research efforts have been made to push the operating wavelength of the SRR-based filters from the microwave to the visible region, the significance of the microwave filters would be tremendously increased if their response characteristics can be dynamically tuned. Therefore, a variety of frequency tuning mechanisms have been reported. Methods include changing unit cell’s effective parameter by varying conductance or inductance^31^,^32^,^33^, configuring constituent material by using phase changeable material as the constituent material^34^,^35^,^36^, and altering geometry through distorting the structure or tilting the conducting elements^37^,^38^,^39^.

Recently, inclusion of liquid metal as active components has opened up new ways to realize stretchable and flexible electronics. By injecting liquid metal into a template, the metal can take on a specific shape to form stretchable electronic devices, such as electrical interconnectors^40^, electrical probes^41^, antennas^42^,^43^,^44^, microelectrodes in microfluidic devices^45^, switchable metamaterial microfluidic platforms^34^, and artificial skin sensors^46^. We previously developed a stretchable single meta-atom in microwave regime, where eutectic gallium-indium (EGaIn: 75.5% gallium and 24.5% indium), a liquid metal at room temperature, was patterned as the SRR structure and embedded inside a stretchable silicone elastomer^39^. As the shape of the liquid SRR was changed via mechanical stretching, the split gap capacitance and the inductance of the resonator were adjusted^39^. While the basic principle of the single stretchable meta-atom was studied in our previous work^39^, it is worthy to note that fabrication, characterization, and application development of flexible and stretchable metamaterials, i.e., arrays of such meta-atoms, have not been achieved. Particularly, it may be difficult in practice to use a single meta-atom to demonstrate applications, such as frequency tunable surface and invisibility cloaking. Therefore, research on the extension from a single meta-atom to metamaterials is critically important. Also, development of metamaterials is relatively application-specific, requiring not only a thorough understanding of the operation of single meta-atoms, but also custom requirements in design, characterization, and quantification. For example, our previously reported single meta-atom was studied in a waveguide setting and behaved as an element in a 2D quasi-periodic system due to the reflective mirror effect of the waveguide walls^39^. However, free space environment is often required to study metamaterials with some practical constraints, such as number of meta-atoms, and layers of metamaterials. Furthermore, the resonance tuning of the reported single meta-atom was realized by shaping the embedded SRR unit in the plane of the host elastomer^39^. However, the effects of integrating multiple layers of metamaterials and changing the spacing between neighboring layers in the direction perpendicular to the surface of the host elastomer are also worthy to be investigated. Furthermore, besides the frequency selection, the extension from a single compliant meta-atom to metamaterials will allow us to study the possible cloaking effect of the flexible metamaterial wrapping on a curved dielectric surface.

In this paper we report a flexible and stretchable microwave meta-skin and its frequency selective and cloaking effects. The meta-skin consists of an array of liquid metal SRR meta-atoms encased inside an elastomer. We demonstrate that by stretching multiple layers of the meta-skins along their surfaces in a planar direction and by changing the spacing between the meta-skin layers in a vertical direction, the meta-skins can perform as a high performance tunable frequency selective surface with a broad tuning range. Furthermore, the meta-skin is able to wrap an interaction object with any arbitrary shapes. We demonstrate that by wrapping it on a dielectric cylindrical rod, a significant scattering suppression or “cloaking” effect is observed. The scattered field from the dielectric rod at different angles is suppressed in a designed frequency region. Therefore, this meta-skin technology is different from traditional stealth technologies that often only reduce the backscattering, i.e., the power reflected back to a probing radar^47^. The present research work tends to fill the gap from the single stretchable meta-atom to the large scale metamaterials by investigating the possibility of realizing resonance tuning of the planar metamaterials and cloaking effect of the curved metamaterials.

## Design and Fabrication

**Figure 1:**
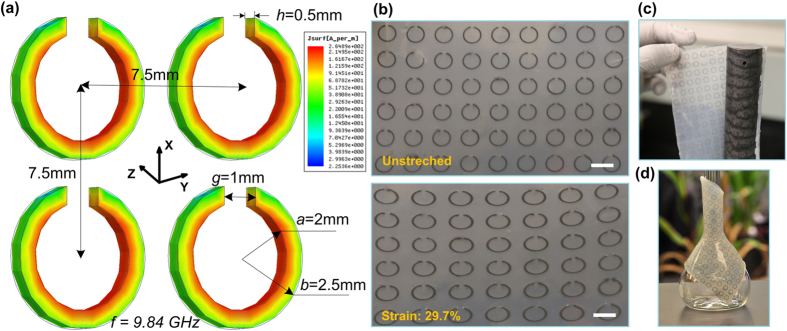
(**a**) Geometry and simulated surface current distribution of the meta-skin. (**b**) Photos of the unstretched and stretched meta-skin. Scale bars represent 5 mm. (**c**) A photo of a 30.48 cm long, 3.175 cm diameter dielectric nylon rod wrapped by the meta-skin. (**d**) Flexibility demonstration with a glass flask wearing the meta-skin.

Figure 1a shows the structure of the proposed SRR array operated in X-Band regime. The device has the inner radius of *a* = 2.0 mm, the outer radius of *b* = 2.5 mm, the thickness of *h* = 0.5 mm, the gap of *g* = 1.0 mm, and the lattice constant of *p* = 7.5 mm. The SRR array is made of EGaIn and encased by a silicone elastomer (Ecoflex). The thickness of the Ecoflex is *d* = 1.45 mm. We conducted EM simulations to estimate a resonance frequency of the array using Ansys High Frequency Structure Simulator (HFSS) software. As shown in Fig. 1a, the SRR array is fixed in the x-y plane, and the magnetic field (**H**) is parallel to the z direction and penetrates through the SRRs, thus exciting a magnetic resonance. With the aforementioned geometrical parameters, the simulated resonance frequency for the SRR array is 9.84 GHz. The surface current distribution at the resonance frequency is shown in Fig. 1a. By stretching the meta-skins (Fig. 1b), the lattice constant, the shape of the SRRs, and the mutual interaction between the resonators will be modulated. Accordingly, the resonance frequency of the meta-skins will be shifted.

**Figure 2:**
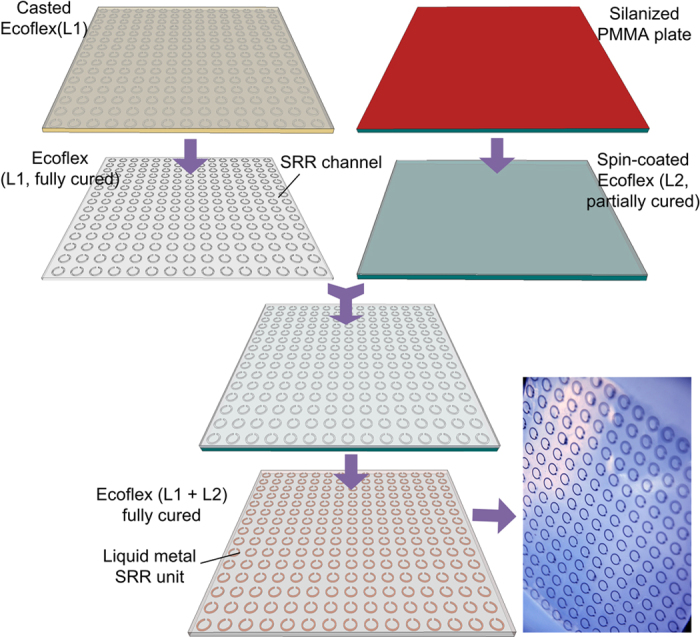
Fabrication process flow for the meta-skin.

We manufactured the proposed meta-skin with 225 identical SRR meta-atoms arranged in 15 columns and 15 rows. Figure 2 shows the fabrication process flow. First, an aluminum master mold with the area of 14 cm × 14 cm was manufactured by using a high precision CNC milling machine. Subsequently, an Ecoflex layer L1 with the thickness of 800 μm was cast upon the master mold and then was fully cured on a hotplate at 60 °C for 30 mins. Simultaneously, another Ecoflex layer L2 was spin-coated on a 3 mm-thick poly(methyl methacrylate) or PMMA plate pretreated with a silane coupling agent. The spin-coated L2 was only partially cured at 50 °C for 1 min. After that, L1 was peeled off from the master mold and then was adhered to L2, followed by baking on the hotplate at 150 °C for 1 min. Thus, the SRR-shaped channels were formed in the elastomer. To inject liquid metal into the channels, an inlet and an outlet were mechanically punched at the two ends of each channel. After the liquid metal was manually injected into the channels by using a syringe (10 mL, Becton-Dickinson) with a needle (20 Gauge), the liquid metal residues were cleaned by a cotton swab dipped with a solution of hydrochloric acid (50%, v/v). Lastly, the whole device was immersed in a prepolymer solution of Ecoflex and then was fully cured at 80 °C for 30 mins. The total thickness of the Ecoflex elastomer was 1.45 mm. The SRRs were located in nearly half the thickness of the elastomer.

## Results and Discussion

**Figure 3:**
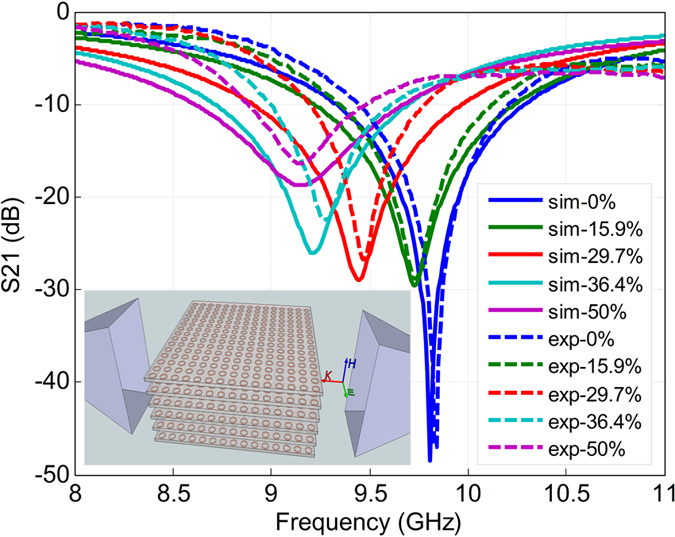
Simulated and experimental transmittance spectra of the tunable 6-layer meta-skins with different stretch ratios of 0%, 15.9%, 29.7%, 36.4%, and 50%. In this experiment the spacing between the neighboring layers is *d* = 3 mm. Inset shows a schematic for the setup.

EM measurements were conducted in free space. Six of the meta-skins were stacked with the initial spacing *d* = 3 mm between neighboring meta-skins. A programmable vector network analyzer (VNA, Agilent E8364) was used to measure spectral responses of the sample. To generate a quasi-plane wave illumination, the meta-skins were placed between two horn antennas (one as a transmitter and the other as a receiver) within the far field regions. As the meta-skins were located in the electric field E-plane of the antenna, the magnetic field **H** could be coupled to the magnetic resonance from the current loop in the SRR (see inset of Fig. 3).

Due to the stretchable feature of the meta-skin, the dimensions of the SRRs can be altered through stretching along different directions. Our previous research showed that the stretch-induced dimensional changes of the SRR can influence the equivalent inductance and capacitance of the SRR, thus shifting its resonance frequency^39^. In the present work, as the multilayers of the meta-skins were stretched along the wave propagation (*k*) direction with the stretch ratio of 0%, 15.9%, 29.7%, 36.4%, and 50%, the resonances of the meta-skins were observed at 9.84 GHz, 9.76 GHz, 9.47 GHz, 9.27 GHz, and 9.15 GHz, respectively. The measurement results are shown in Fig. 3 with dashed lines. To verify the measured results, an HFSS-based full wave EM simulation was carried out by applying the periodic boundary condition on the SRR units. The simulation results with different stretch ratios are shown in Fig. 3 with solid lines. The simulated and experimental results achieved a good agreement in the trend of shifting resonance frequency. The minor difference in the resonance frequency and bandwidth may be attributed to the accuracy of the model.

**Figure 4:**
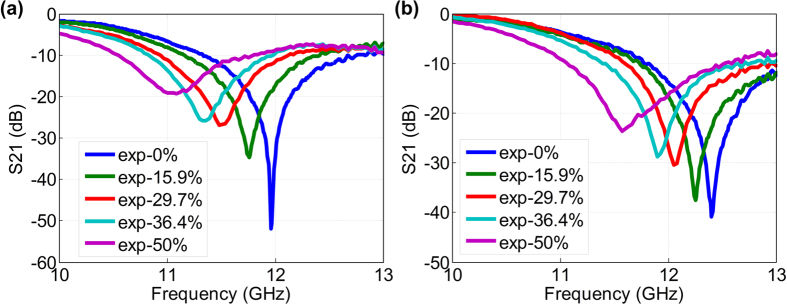
Experimental transmittance spectra of the tunable 6-layer meta-skins with different stretch ratios of 0%, 15.9%, 29.7%, 36.4%, and 50% for two different spacing between neighboring layers: *d*_1_ = 13 mm (**a**) and *d*_2_ = 17 mm (**b**).

By changing the spacing d between two neighboring layers, the resonance of the 6-layer meta-skins could also be tuned. Here, the spacing was defined by inserting foams (relative permittivity close to one). Figure 4a,b show the spectral responses of the meta-skins to different stretching levels for the spacing of *d*_1_ = 13 mm and *d*_2_ = 17 mm, respectively. As the meta-skins moved farther away from each other, the mutual inductance between the resonators in the neighboring layers reduced^48^. Consequently, an increase in resonance frequency of the meta-skins is expected. Indeed, for the unstretched sample, the resonance frequency was shifted from 9.84 GHz to 11.9 GHz as the vertical spacing increased from 3 mm to 13 mm. As we further increased *d* to 17 mm, the resonance frequency was shifted to 12.4 GHz. Similarly, by stretching the multilayer metal-skins along their surfaces in the horizontal direction, the resonance frequency was also observed to move towards lower frequencies. Therefore, by varying the spacing between the meta-skins in the vertical direction and stretching the metal-skins in a planar direction, the resonance frequency tuning range of the meta-skins can be largely broadened.

**Figure 5:**
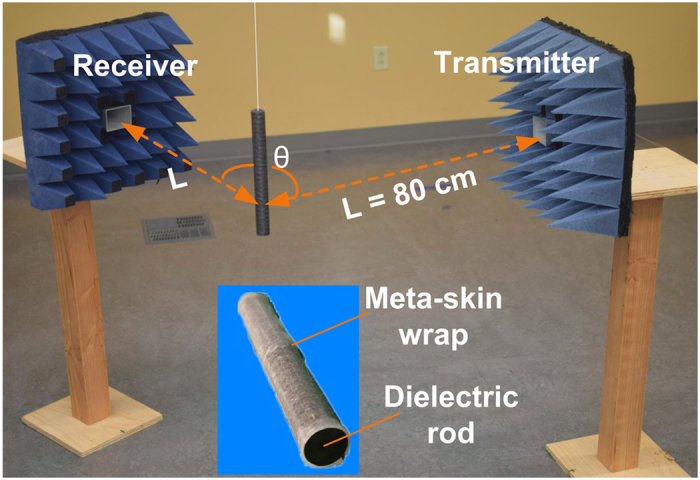
Experimental setup for measuring scattering from the meta-skin wrapped nylon rod (inset).

The fully flexible nature of the meta-skin makes it possible to wrap on an interaction object with any arbitrary shapes (Fig. 1d). Here, we used a single layer of meta-skin to wrap on a 30.48 cm long, 3.175 cm diameter dielectric nylon rod (dielectric constant: 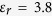; Fig. 1c). We investigated how this wrapping material could influence the scattered field from the rod. The far-field measurement was thus conducted to measure scattering strength from a bare nylon rod, a nylon rod wrapped by a pure Ecoflex sheet, and a nylon rod wrapped by the meta-skin. In the measurement setup (Fig. 5), the sample hangs from a cotton thread at a designated origin to minimize unwanted scattering signals from the support constructs. The two horn antennas were placed at an equal distance of *L* = 80 cm from the sample. This ensured that the object was in the far-field region (according to the far-field condition 2*D*^2^/*λ*, where *D* = 9.8 cm is the diagonal of the horn antenna and *λ* = 3 cm is estimated from the center operating frequency). The transmitter antenna was fixed during the measurement, while the receiver antenna was moved around the sample to receive scattering signals from different angles θ with respect to the transmitter. The two horn antennas were inset into the EM absorbing material to minimize the scattering background. The aforementioned VNA was used to measure scattering parameters between the two antennas. The objective azimuthal bistatic measurements were conducted to obtain the s-parameter for further data processing. The method of deriving the scattered field from the dielectric sample is described in the Method section.

**Figure 6:**
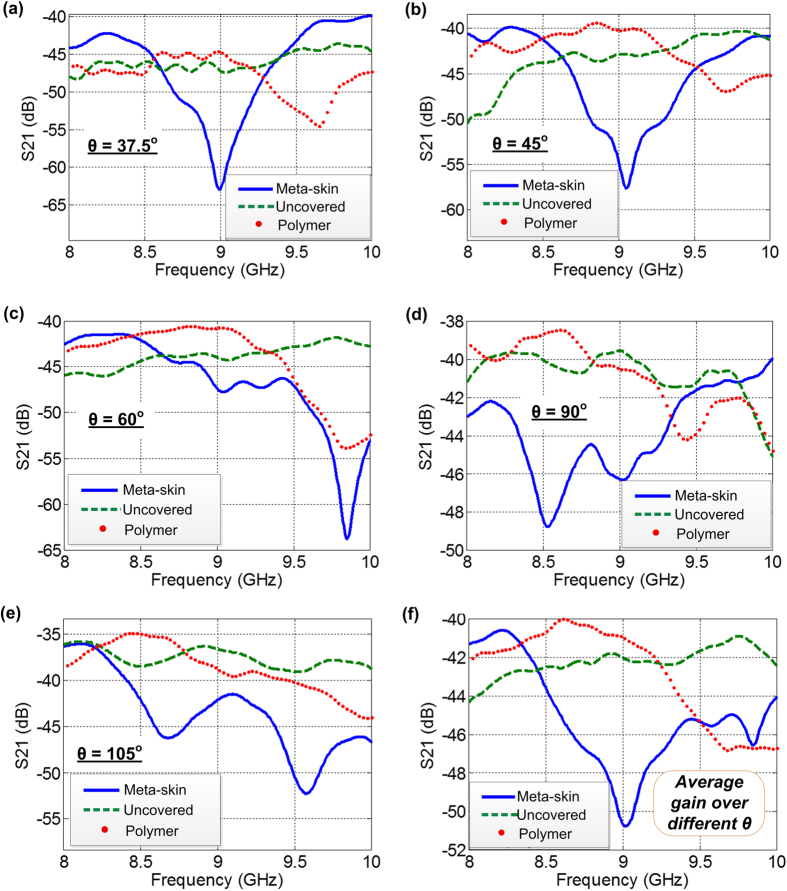
Measured scattering gain for the bare dielectric rod (green – uncovered), the rod wrapped with the Ecoflex polymer (red – polymer), and the rod wrapped with the metal-skin (blue – meta-skin) at the angles of *θ* = 37.5^o^ (**a**), 45^o^ (**b**), 60^o^ (**c**), 90^o^ (**d**), and 105^o^ (**e**). The average scattering gain over the different angles is given in (**f**).

The post-processed scattering gains for the meta-skin covered, the polymer covered, and the uncovered rods are presented in Fig. 6a–e. The results show that the scattering gain from the meta-skin covered rod was significantly reduced in the frequency band from 8 GHz–10 GHz at five different measurement angles *θ* = 37.5^o^, 45^o^, 60^o^, 90^o^, and 105^o^. Specifically, compared with the uncovered case, at *θ* = 37.5^o^ the meta-skin was able to suppress the scattering gain in over 33% of frequency band between 8–10 GHz. At other angles the suppression effect of the meta-skin is more significant and the scattering grain was suppressed in over 70% of the same frequency range. The largest suppression of 20 dB was found at around 9 GHz at 37.5^o^. The overall scattering suppression effect of the meta-skin is illustrated by averaging the scattering gain with different angles (Fig. 6f). It is observed that the meta-skin was able to suppress the scattering gain by about 75% in the band of 8–10 GHz. The scattering suppression is mainly attributed to the cloaking effect of the embedded SRRs around the designed frequency, where the destructive interference between the resonance of the SRRs and the scattering from the dielectric rod occurred. We also noted that the scattering gain spectra of the meta-skin wrap in Fig. 6 do not have exactly the same resonance frequency as the transmittance spectra of the multilayer meta-skins in the unstretched state in Fig. 3. The factors below may be attributed to this observation: first, the scattering suppression gain was measured at different angles, which actually is angle-dependent due to different phases of multiple reflections and interactions over the interfaces; second, only one layer of the meta-skin was coated on the surface of the nylon rod with the dielectric constant of 3.8, while multiple layers of the meta-skins were used in the frequency selective surface application and spaced by foam with the dielectric constant of close to one.

The whole meta-skin remained fully functional without fatigue or cracking after repeated measurements. This is because the liquid metal SRRs can flow and reshape responding to applied strains. Besides the single circular SRR, many other magnetic resonance structures may be used in the meta-skin setting to realize frequency selection and scattering suppression^49^. Furthermore, in addition to the magnetic resonators, liquid metal-based electric resonant structures, such as wires, can be integrated into the same elastomer. This will make it possible to achieve negative index for cloaking applications. Moreover, other different stretchable and flexible dielectric host media could be used to embed these liquid metal-based resonant structures. This will provide us with more flexibility to control loss tangent of the meta-skin. In the microwave frequency regime the dielectric losses are dominant and different substrate dielectric materials can affect the loss tangent. For higher frequencies, such as terahertz, as the ohmic losses become significant, other types of liquid metal or conducting materials are required to form the resonating units.

## Conclusions

A stretchable and wearable microwave meta-skin was developed by embedding an array of liquid metal SRRs into a highly stretchable elastomer. We demonstrated the strong ability of the meta-skin to tune the resonance of the frequency selective surface and to suppress the scattering from the curved surface of a dielectric material along different directions. By combining the planar stretching and the vertical spacing, the resonance frequency of the multilayer meta-skins was tuned from 9.15 –12.38 GHz. By wrapping a finite-length dielectric rod with the meta-skin, the scattering from the surface of the rod was suppressed by about 75% in 8–10 GHz. It is believed that the present meta-skin technology will find many applications in EM frequency tuning, shielding, and scattering suppression.

### Methods of deriving scattered field from dielectric sample

**Figure 7:**
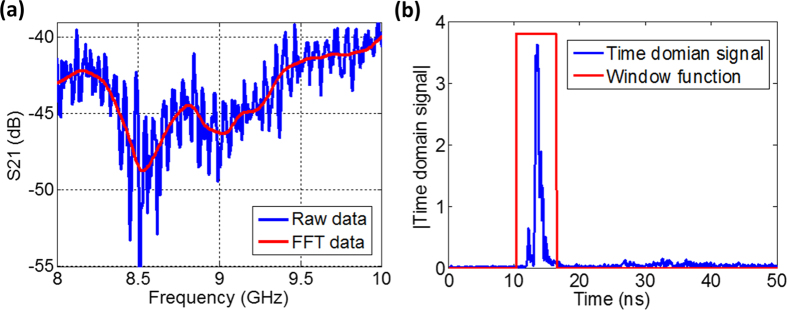
(**a**) Raw and processed scattering gain for the meta-skin coated nylon rod at the angle of *θ* = 90^o^. (**b**) Gated time domain signal.

The scattered field was obtained by subtracting the incidence field (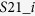) from the total field (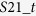). This operation can not only derive the field scattered from the sample, but minimize clutters from background in the experiment. Two consecutive measurements were conducted. First, the sample was placed in the designated position and 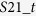 was measured. Then, the sample was removed and 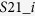 was recorded. The original scattering parameter for the scattered field can be expressed as 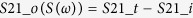. It should be noted that because the real testing environment was complicated, the *S*(*ω*) derived from subtracting the incidence field from the total field may not entirely remove the clutter component from the objective signal. As shown in Fig. 7a, the spectral response of *S*(*ω*) (solid blue line) contains complex stray signal. To efficiently remove the clutters, we applied fast Fourier transform for the post processing, where a Gaussian window function (*G*(*ω*)) was adopted due to its high resolution in time domain. By multiplying *G*(*ω*) by *S*(*ω*), a new signal in frequency domain was generated, denoted as *H*(*ω*). To reveal the signal response in the time domain, an inverse fast Fourier transform (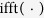) was implemented over *H*(*ω*) to generate *h*(*t*). Further, *h*(*t*) was multiplied by a designated rectangular window function *w*(*t*). In time domain, the signals from clutters were late arrived. Applying the rectangular window function in time domain allows filtering out the scattering from the clutters. The gated time domain signal is shown in Fig. 7b. Finally, the fast Fourier transform 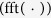 was implemented over this processed time domain signal (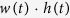), yielding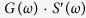, where 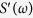 solely represents the interaction with the sample in the frequency domain. This entire process can be expressed


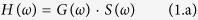



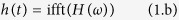



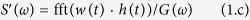


Using Equation 1, we obtained the scattered field from the aforementioned three different samples. The processed scattered field for the sample covered by the meta-skin is shown in Fig. 7a with red solid line. Compared to *S*(*ω*), 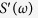
becomes smooth after the removal of the clutter from *S*(*ω*), while the scattering information still is contained.

## Additional Information

**How to cite this article**: Yang, S. *et al.* From Flexible and Stretchable Meta-Atom to Metamaterial: A Wearable Microwave Meta-Skin with Tunable Frequency Selective and Cloaking Effects. *Sci. Rep.* **6**, 21921; doi: 10.1038/srep21921 (2016).
